# Glioblastoma Stem-Like Cells Are More Susceptible Than Differentiated Cells to Natural Killer Cell Lysis Mediated Through Killer Immunoglobulin-Like Receptors–Human Leukocyte Antigen Ligand Mismatch and Activation Receptor–Ligand Interactions

**DOI:** 10.3389/fimmu.2018.01345

**Published:** 2018-06-18

**Authors:** Heleen Neeltje Haspels, Mohummad Aminur Rahman, Justin Vareecal Joseph, Andrea Gras Navarro, Martha Chekenya

**Affiliations:** Department of Biomedicine, University of Bergen, Bergen, Norway

**Keywords:** glioblastoma, differentiation, NK-cells, KIR-HLA interactions, cancer stem-like cells, natural killer cells, killer immunoglobulin-like receptors–human leukocyte antigen ligand mismatch

## Abstract

Glioblastoma (GBM) is the most aggressive brain malignancy in adults, where survival is approximately 14.6 months. Novel therapies are urgently needed and immunotherapy has hailed a new dawn for treatment of solid tumors. Natural killer (NK) cells may be amenable therapeutic effectors against heterogeneous GBM, since they also do not require co-stimulation and antigen specificity. However, it is unclear how culture media routinely used in pre-clinical studies affect GBM cell responses to NK-mediated cytotoxicity. We hypothesized that the culture medium would affect GBM cell phenotype, proliferation, and responses to NK cytotoxicity. We investigated in paired analyses *n* = 6 patient-derived primary GBM cells propagated in stem cell or serum-containing medium for morphology, proliferation, as well as susceptibility to NK cytolysis and related this to expression of surface and intracellular lineage markers, as well as ligands for NK cell activating and inhibitory receptors. We genotyped the GBM cells for human leukocyte antigen (HLA) as well as the killer immunoglobulin-like receptors (KIR) of the *n* = 6 allogeneic NK cells used as effector cells. Culture in serum-containing medium induced a switch in GBM cell morphology from suspension neuropsheres to adherent epithelial–mesenchymal-like phenotypes, which was partially reversible. The differentiated cells diminished expression of nestin, CD133 (prominin-1), and A2B5 putative glioma stem-cell markers, attenuated growth, diminished expression of ligands for activating NK cell receptors, while upregulating class I HLA ligands for NK cell inhibitory receptors. When maintained in serum-containing medium, fewer GBM cells expressed intercellular cell adhesion molecule-1 (ICAM-1) and were less susceptible to lysis by NK cells expressing α_L_β_2_ integrin receptor (LFA-1), mediated through combination of inhibitory KIR–HLA ligand mismatch and diminished activation receptor–ligand interactions compared to cells maintained in stem cell media. We conclude that development of preclinical immunotherapy strategies against GBM should not use cells propagated in serum-containing media to avoid misinterpretation of potential therapeutic responses.

## Introduction

Glioblastoma (GBM) is the most frequent malignant brain tumor in adults (1). Although the age-adjusted incidence is relatively low at 3.2/100,000 people in Western countries including United States (2), patients’ median survival rate is only 14.6 months, notwithstanding aggressive multimodal treatment consisting of surgery, radiotherapy, and temozolomide (TMZ) chemotherapy (3). When combined with TMZ, a novel tumor-treating field device, Optune^®^, extended survival to 20.9 months (4) and now represents an additional treatment modality. Nevertheless, approximately 5.1% of patients are alive 5 years post diagnosis (3), underscoring the dire and unmet need for more effective treatment.

Immunotherapy has emerged as a successful treatment of choice for several cancers, particularly with the advent of checkpoint blockade and T cell receptor engineering (5–7). This treatment modality has yet to reap benefit for brain tumor patients, largely due to challenges posed by the blood–brain barrier, tumor heterogeneity, and the highly immunosuppressive solid tumor contexture (8–11). Although high T cell infiltration to the brain tumor is of prognostic benefit, effector activity is insufficient to ameliorate tumor progression (9, 12, 13). However, among cytotoxic lymphocytes, natural killer (NK) cells may be more suitable as therapeutic effectors against highly heterogeneous tumors such as GBM, since unlike T and B lymphocytes, they do not possess rearranged V(D)J receptors and are not restricted by major histocompatibility complex (MHC)-bound antigen presentation. They broadly recognize pathogen infected and transformed cells without particular antigen specificity or requirement for co-stimulation. Instead, NK cells express an exquisite repertoire of inhibitory killer immunoglobulin-like receptors (KIR) for MHC/human leukocyte antigen (HLA), that when ligated to cognate self-HLA, dampen their effector functions to maintain self-tolerance and avoid autoreactivity, while simultaneously killing targets lacking the self-molecules (14, 15).

Structurally, KIRs harbor two or three extracellular-C2-type domains with long (KIR2DL or KIR3DL) or short (KIR2DS or KIR3DS) tails and possess inhibitory or activating capabilities, respectively (16–18). In particular, KIR2DL1 exclusively recognizes the HLA-C2 group of ligands possessing lysine residues at position 80 of the α-helix and is considered the strongest inhibitory KIR (19, 20), whereas KIR2DL2 and KIR2DL3 allelic forms recognize the HLA-C1 group of ligands with asparagine residues at position 80 and have respectively, intermediate and moderate inhibitory capabilities. KIR3DL1 interacts with Bw4-containing HLA-B alleles, KIR3DL2 recognizes HLA-A3 and HLA-A11 allotypes, while KIR2DL4 binds non-classical HLA-G. NK cells become “licenced” and fully functional effectors after recognition of self-HLA alleles by corresponding inhibitory KIR during development (21–23). NK cells’ education on self-HLA allows rapid cytolysis of distressed or malignant cells lacking the class I HLA ligand cognate to inhibitory KIRs expressed (24) (KIR–HLA ligand mismatch).

In addition, NK cells express CD94/NKG2A heterodimers that recognize non-classical HLA-E as ligand. Encounter with cells lacking surface HLA molecules, “missing self” (14, 15), relinquishes the NK cells’ inhibitory signals rendering them directly cytotoxic to target cells or lethal through effects of cytokines, such as interferon gamma (IFNγ) or tumor necrosis factor alpha. Through these cytokines, NK cells have the potential to transform the anti-inflammatory solid tumor microenvironment to a pro-inflammatory, thus shaping the amplitude of the adaptive immune response (25). NK cells also express a diverse set of activating receptors including NKG2D (26), that recognizes ligands expressed on cancer cells due to genotoxic stress, e.g., MHC chain-related antigens (MICA/B) and the UL16-binding proteins (ULBPs) (27, 28), as well as natural cytotoxicity receptors (NCRs) NKp30 and DNAM-1 that recognize, respectively, B7-H6 and CD112. Ultimately, it is the threshold balance between cooperative or antagonizing activation versus inhibition signals that determine NK cell cytotoxicity and or cytokine production in their effector phase (29, 30).

Early studies that deemed the central nervous system immune privileged might have stymied progress in evaluating the amenability of GBM to cellular immunotherapy. Subsequent studies perhaps overstated the impassability of the blood–brain barrier (BBB) (31). It is now accepted that the BBB is variably disrupted in GBM patients, permitting immune cell trafficking and that intracerebral antigens extravasate through the cerebrospinal fluid to the subarachnoid space to draining cervical lymph nodes (32, 33). Moreover, GBM cells were previously erroneously considered refractory to NK cell lysis (34, 35) as these studies utilized resting, unactivated NK cells or NK cell lines with limited cytolytic activity. Furthermore, GBM cell lines are routinely used as the initial preclinical standard for exploring the biology and therapy responses of human tumors (36). It is increasingly clear that their immunological phenotypes after long-term *in vitro* passage do not resemble those of the corresponding primary tumor. Moreover, numerous genetic aberrations arise *de novo* in cell lines that may not be present in the mother tumor (37). These anomalies can lead to misinterpretations of biological behavior and interpretations of therapy responses of the tumors studied. Not only the number of passages is responsible for this but ostensibly, differences arise as a consequence of adaptation to presence of animal serum in the culture medium over time.

Previous studies demonstrated that serum-cultured cells lose their self-renewing capabilities and terminally differentiate (36, 38, 39). Their gene expression profiles no longer resemble the primary GBMs and sometimes they even lose their ability to form tumors in animals. In contrast, primary GBMs propagated in stem cell medium retain their similarity to normal neural stem cells (36) evidenced by their ability to form neurospheres *in vitro*, retain potential for self-renewal and similar gene expression profiles to their parental tumors. We do not know however, whether functionally, these cell culture methods induce differences in responses to the new immunotherapy paradigms in short-term GBM cells. Thus, we hypothesized that the culture medium might have an effect on primary GBM phenotype, cell proliferation, and response to NK cytotoxicity and investigated this in paired samples maintained in stem cell-versus serum-containing culture medium.

## Materials and Methods

Patient-derived GBM samples were obtained from routine surgical resections undertaken at Haukeland University hospital, Bergen Norway. Ethical approval for collection of tissue and blood samples was obtained from the regional committee of Western Norway (REKvest 2013/720) and The Norwegian Data Protection Agency. Ethical approval was also obtained (REKvest 2014/588) to collect blood from healthy blood donors from the Blood Bank and Transfusion Unit, Haukeland University Hospital. Patients and donors gave their informed consent to specimen collection for research.

### Patient-Derived GBM Cell Culture

The patient derived primary GBM cells P3, 2012-018, BG5, BG7, GG1, and GG9 were generated as previously described (40, 41) and propagated in NeuroBasal^®^ medium (NB, Invitrogen Hämeenlinna, Finland) supplemented with 1% v/v GlutaMAX^®^ (Invitrogen), 2% v/v B-27^®^ (Supplement Minus Vitamin A, Invitrogen), 1% v/v Penicillin/Streptomycin (PS), 20 ng/ml recombinant human epidermal growth factor (EGF, PeproTechc SE, Stockholm), and 20 ng/ml recombinant human basic fibroblast growth factor (FGF, PeproTech SE, Stockholm). Alternatively, they were cultured in Dulbecco’s modified eagle medium (DMEM, Sigma-Aldrich, St. Louis, MO, USA) supplemented with 10% fetal bovine serum, non-essential amino acids, 100 U/ml PS and 400 μM l-glutamine (all Cambrex, East Rutherford, NJ, USA) (complete medium) as previously described (41) at 37°C in a humidified atmosphere of 5% CO_2_. They were passaged when 80% confluent and only cells in the exponential phase and within passage 32 were used for the experiments, Table S1 in Supplementary Material.

### GBM De-Differentiation and Neurosphere Measurement

Neurospheres were dissociated into single cells using accutase (Sigma-Aldrich), and seeded at a density of 1 million cells/well in poly l-Lysine coated T75 flasks in 15 ml of serum-containing medium, which was replenished every 3 days. After 14 days of culture in differentiation medium, the cells were harvested, washed in PBS and counted. Protein lysates were extracted, while the rest of cells were reseeded at a density of 10,000 cells/well in stem cell medium in 6-well plate or 10, 20, or 40 cells/well in 96-well plate in final volume of 100 µl stem cell medium. At day 4 fresh stem cell medium was added and after 15 days the images of spheroids were captured on Motic AE31E microscope and diameters (μm) of 50 neurospheres from each tumor before and after de-differentiation were measured by the Motic Image Plus 3.0 software (Speed Fair Company, Ltd., Hong Kong). The diameters of original undifferentiated primary spheroids were statistically compared to spheroids generated after de-differentiation of the differentiated cells.

### Immunofluorescence

Cells were cultured on poly l-lysine (Sigma-Aldrich) coated cover slips and fixed for 10 min using 4% formaldehyde. After washing three times with PBS, the cells were permeabilized with 0.1% Triton (Sigma-Aldrich) in PBS prior to blocking for 1 h with PBS+ 1% Tween20 (Sigma-Aldrich), 2% BSA (Brunschwig), and 1:50 normal goat serum (Dako). The cells were incubated with primary antibodies at room temperature for 2 h, followed by incubation for 1 h with corresponding secondary antibodies, Cy3 (1:400 millipore) or Alexa 488 (1:400 Invitrogen). Hoechst 1:500 counter staining for 5 min was undertaken and coverslips mounted using Kaisers glycerin (Merck).

### Western Bloting

Cells lysates were prepared in Kinexus lysis buffer (20 mM MOPS, 5 mM EDTA, 2 mM EGTA, 30 mM NaF, 0.5% Triton X, 1 mM PMSF, pH 7.2), protease inhibitor (cocktail tablet, Roche; Basel, Switzerland), and phosphatase inhibitor (cocktail tablet, Roche). 20 µg protein was run on SDS/PAGE with NuPage precast 4–12% gradient gels (Invitrogen; Carlsbad, CA, USA). Blots were incubated 1 h at room temperature with primary antibodies followed species specific secondary HRP-conjugated antibodies. Chemilumescence detection was performed with Super Signal West Femto Maximum Sensitivity Substrate (Thermo Fisher Scientific) on the LAS-3000 (Fujifilm Medical Systems Inc.; Stamford, CT, USA).

### Population Doubling Time (PDT)

To determine the PDT, 5 × 10^4^ GBM cells were seeded in DMEM or NB medium in triplicate and counted after 24, 48, 72, and 96 h using a BRAND^®^ counting chamber BLAUBRAND^®^ Bürker-Türk. Each condition was repeated in at least three independent experiments. PDT was calculated by using the following equation:

$$\text{Population doubling time }(\text{hours})=\frac{\text{Incubation time }(\text{hours})\times \text{LOG}(2)}{\text{LOG }(\frac{\text{Number of cells at the end of incubation time}}{\text{Number of cells at the beginning of incubation time}})}.$$

### Peripheral Blood Mononuclear Cells (PBMC) Isolation

Peripheral blood was collected into BD Vacutainer^®^ CPTTM Cell Preparation Tube with Sodium Citrate (BD Biosciences, Franklin Lakes, NJ, USA). PBMC were isolated from whole blood using SepMate 50-ml tubes with a high-density polyethylene membrane (Greiner Bio-One, Frickenhausen, Germany) and lymphocyte separation medium (Lonza, Lysaker, Norway), centrifuged for 10 min at 1,200 *g*. Cells were cryopreserved and frozen down in RPMI 1640 medium (Invitrogen), 25% v/v human serum (Sigma-Aldrich), 7% v/v DMSO in liquid nitrogen.

### NK Cell Isolation and Culture

Natural killer cells were purified from the PBMCs of healthy blood donors by negative selection on magnetic columns (NK Cell Isolation Kit, Miltenyi Biotech, Germany), according to manufacturer’s protocol as previously described (42). NK cells were cultured in 48-well plate at 37°C and 5% CO_2_ for 2 weeks in Stem Cell medium (SC, Cell Genix, Freiburg, Germany) supplemented with 10% v/v fetal cow serum (FCS), 500 U/ml IL-2 and activation microbeads of NK Cell Activation/Expansion Kit (Miltenyi Biotech) according to manufacturer’s protocol. From day 4, fresh medium supplemented with 500 U/ml IL-2 was replenished daily, and after 14 days, the NK cells were used as effectors in cytotoxicity assays.

### Genotyping KIR Receptors and HLA Ligands

DNA was isolated from GBM patients’ tumor and blood, as well as donors’ whole blood using the DNeasy Blood & Tissue Kit (QIAGEN, Germantown, MD, USA), according to the manufacturer’s protocol and concentration measured by a Nanodrop 1000 Spectrophotometer (Thermo scientific). KIR typing was performed using the KIR Typing kit (Miltenyi Biotec), according to the manufacturer’s protocol. HLA typing to two-digit resolution was performed by ProImmune (Oxford, UK).

### Flow Cytometry Phenotyping

Glioblastoma cells (0.25–1 × 10^6^ cells) and NK cells (0.5–1 × 10^6^ cells) were stained in FACS tubes with the relevant previously optimized concentrations of primary Abs for surface and intracellular antigens (Table S1 in Supplementary Material) as previously described (42). After staining for surface antigens, GBM cells were fixed and permeabilized using Cytofix/Cytoperm Fixation/Permeabilization Solution Kit (BD Biosciences, Trondheim, Norway) and stained for intracellular antigens. For cytotoxicity assays, NK effector cells were labeled with 5 mM 5(6)-Carboxyfluorescein Diacetate N-succinimidyl ester (CFSE, Sigma-Aldrich) for 20 min at 37°C protected from light and mixed for every 5 min. Effector cells were then co-cultured with target GBM cells at iterative effector:target (E:T) ratios of 5:1, 10:1, and 20:1 for 4 h at 37°C and 5% CO_2_ in RPMI 1640 medium (Sigma-Aldrich, St. Louis, MO, USA) supplemented with 10% v/v FCS (Sigma-Aldrich), 50 U/ml penicillin–streptomycin (Lonza), and 1 mM Hepes with 0.85% NaCl (Lonza). After washes in PBS containing 0.5% BSA (Miltenyi Biotec) cells were labeled with Live/Dead Fixable Near-IR Dead Cell Stain Kit (Invitrogen). GBM, target cells were identified as CFSE^−^, and effector, NK cell were identified as CFSE^+^, thus dead target cells were CFSE^−^ and Live/Dead^+^. Spontaneous death was defined as the proportion of dead cells in the negative control, and this value was subtracted from the proportion of dead target cells co-cultured with effector cells (Figure 4G). Fluorescent minus one (FMO) controls were used for each channel. Data were acquired on the LSR Fortessa™ (BD Biosciences) and analyzed using FlowJo, version 10 (Tree Star Inc.; Ashland, OR, USA).

### Statistical Analysis

When comparing more than two groups with one dependent variable, one-way ANOVA was used; otherwise two-way ANOVA was used to analyze data with two or more dependent variables compared in two or more groups. Bonferroni *post hoc* test was used to correction for multiple comparisons. *P*-values <0.05 were considered statistically significant (shown as *P < 0.05, **P < 0.01, ***P < 0.001, and ****P < 0.0001). Descriptive statistics are reported as mean ± SEM of at least three independent experiments, unless otherwise stated. All statistical analyses were performed with the Prism statistical software, version 5.0 (GraphPad, La Jolla, CA, USA).

## Results

### Culture Medium Impacts Patient-Derived GBM Cells’ Morphology and Phenotype

**Figure 1:**
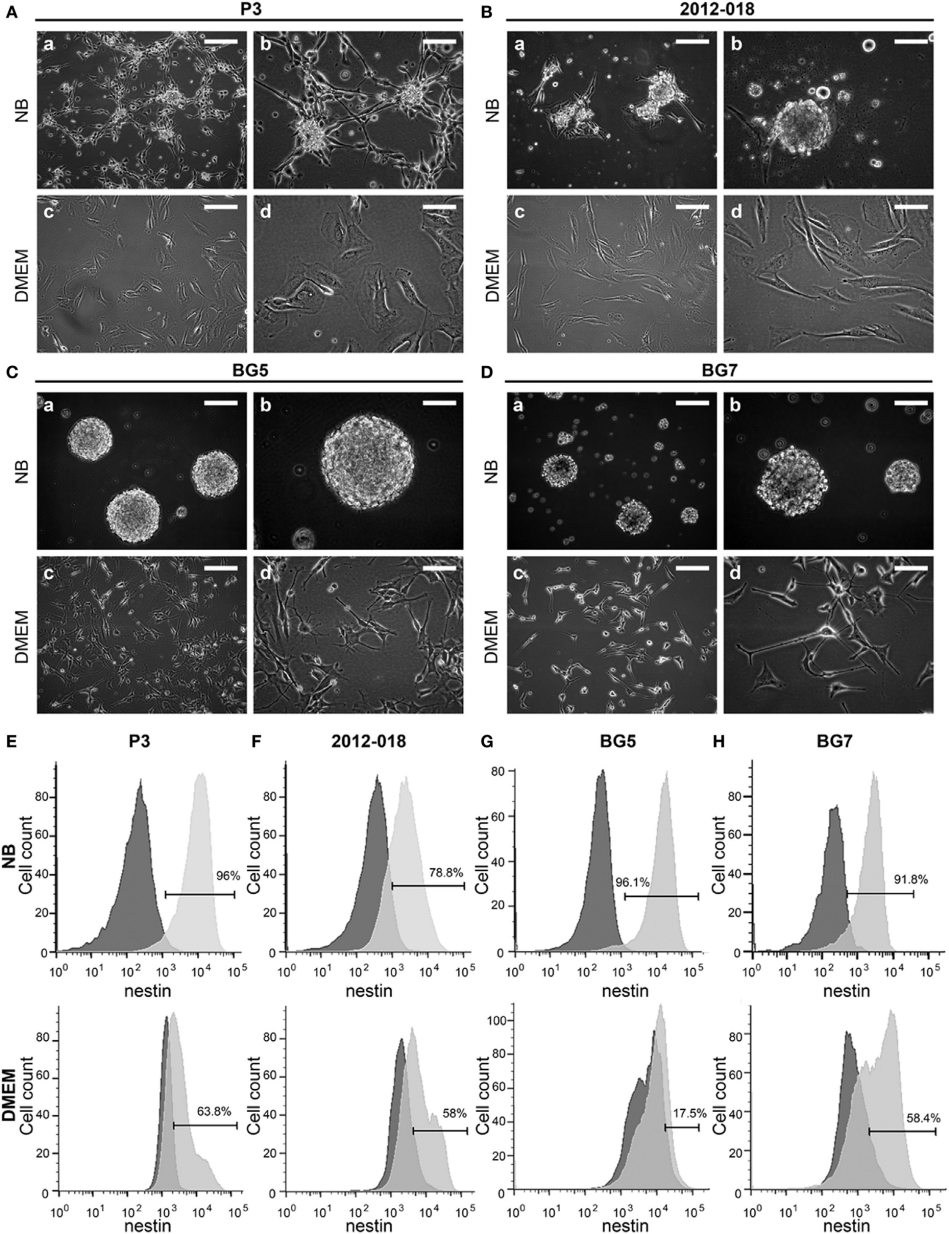
Culture medium impacts patient-derived glioblastoma (GBM) cells’ morphology and phenotype. Cell morphology of **(A)** P3, **(B)** 2012-018, **(C)** BG5, and **(D)** BG7 GBM cells cultured in NB **(a)** magnification 100×, scale bar 50 µm, **(b)** magnification 200×, scale bar 50 µm; and in Dulbecco’s modified eagle medium (DMEM) **(c)** magnification 100×, scale bar 50 µm, (d) magnification 200×, scale bar 50 µm. Histograms showing intracellular expression of nestin on **(E)** P3, **(F)** 2012-018, **(G)** BG5, and **(H)** BG7 GBM cells cultured in NB (upper histograms) and in DMEM (lower histograms). Dark gray histograms—fluorescent minus one control and light gray histogram—nestin.

To test the hypothesis that culture of GBM cells in serum-containing or serum-free stem cell media might influence morphology, phenotype, and growth, we used low passage biopsy-derived GBM cells from patients P3, 2012-018, BG5, BG7, GG1, and GG9 (Table S2 in Supplementary Material). P3 cells propagated in stem cell medium generated phase bright neurospheres with some adherent cells (Figure 1Aa,b). However, when propagated in serum-containing media, the cells grew exclusively as adherent monolayers and exhibited an epithelial-like morphology (Figure 1Ac,d). GBM cells from patient 2012-018 formed partial neurospheres with few adherent cells in stem cell medium (Figure 1Ba,b) and were fully adherent in serum-containing medium (Figure 1Bc,d). BG5 and BG7 GBM cells grew exclusively as neurospheres in stem cell medium (Figure 1Ca,b, and Figure 1Da,b, respectively) and adhered to the substratum as mixed cell types with mesenchymal-astrocytic morphology in serum-containing medium (Figure 1Cc,d and Figure 1Dc,d, respectively). Taken together, a switch in GBM morphology from suspension neuropsheres to adherent epithelial–mesenchymal occurred after maintenance in serum-containing medium.

### Serum-Containing Medium Differentiates GBM Cells

To investigate whether the morphological changes were result of differentiation, we phenotyped the cells for various surface and intracellular markers of immaturity and differentiation. As expected due to their glial lineage, a high proportion of the GBM cells consistently expressed glial acidic fibrillary protein (GFAP), Table 1. However, the greatest density of the intermediate filaments was after culture in serum-containing medium (Table S3 in Supplementary Material). Nestin was consistently upregulated in all GBM cells maintained in stem cell medium (Figures 1E–H; Table 1) albeit with variable intensity (Table S3 in Supplementary Material). In contrast, the proportion of cells expressing vimentin intermediate filaments varied depending on the tumor and culture medium (Table 1). CD15, a glycoprotein whose expression is reported enriched in glioma stem-like cells (43) was only modestly expressed in all GBM cells regardless of culture medium (Table 1). Likewise, the proportion of CD133 (Prominin-1) expressing cells varied depending on tumor and culture medium (Table 1), although the density of the glycoprotein on the surface was consistently elevated after culture in serum-containing medium (Table S3 in Supplementary Material). Lower fractions of GBM cells expressed A2B5 when propagated in stem cell medium (range 23.2–63.3%) except for BG5 where 90.4% of these cells expressed it (Table 1). In contrast, when the cells were maintained in serum-containing medium, A2B5 expression density was upregulated in all GBM cells (Table S3 in Supplementary Material). Increased GBM cells maintained in stem cell medium expressed the intercellular cell adhesion molecule-1 (ICAM-1) whose expression were markedly reduced after culture in serum-containing medium, with the exception of BG7 (Table 1). Taken together, primary GBM cells in serum-containing medium differentiate, indicated by diminished nestin, increased GFAP intensity, and reduced expression of the putative glioma stem-cell markers, albeit with tumor and culture medium dependent variability.

**Table 1:** Phenotype of glioblastoma cells (% of cells expressing the markers) in NB and Dulbecco’s modified eagle medium (DMEM) media.

	NB				DMEM			
	P3	2012-018	BG7	BG5	P3	2012-018	BG7	BG5
Glial acidic fibrillary protein	99.1	99	98	100	99.9	99.3	91.4	92
Vimentin	42.9	49.5	97.1	83.9	66.2	70.7	17.2	25.3
Nestin	96	78.8	91.8	96.1	63.8	58	58.4	17.5
CD15	0	0.22	0.34	2.61	0	0.3	1.05	0.2
CD133	11.9	12.2	1.53	2.5	2.09	4.53	40.2	6.97
A2B5	63.3	23.2	39.6	90.4	98.4	91.7	66.6	48.1
CD31	13.4	9.12	0.45	40.2	31.1	38	79.4	31
ULBP-1	41.3	57.5	18.6	7.87	12.1	16.8	2.96	8.93
ULBP-2/5/6	91.7	98.2	48.6	64.4	99.7	87.9	28.2	17
ULBP-3	94.7	95.1	66.2	84.3	90.4	43.9	30.2	12
MICA	85.1	95.1	40.1	58.2	76.7	92.8	14.6	34.9
MICB	11.6	10.1	9.2	17.7	4.25	9.15	4.79	4.11
B7-H6	19.1	18.9	4.41	5.51	6.7	8.35	6.25	4.11
Intercellular cell adhesion molecule-1	18.1	52.8	16.6	35.4	7.68	49.8	26.7	21.6
CD112	95.6	96.1	91.7	93.5	47.5	54.1	37.1	82.8
Human leukocyte antigen (HLA)-G	98.1	99.8	10.5	99.4	99.9	99.3	81	95.6
HLA-E	99.7	99.8	18.5	99.9	100	99.9	99	96.9
HLA-A, B, and C	67.9	88.9	92.8	97.9	99.8	99.7	97	94.3
HLA-DR, DP, and DQ	64.9	95.6	8.85	87.7	98.4	60.7	19.7	27.4
HLA-A3	–	99.2	0.9	96	0	99.4	0.8	72.9
HLA-Bw4	97.2	99.5	–	–	99.4	98.9	–	–

### GBM Differentiation in Serum-Containing Medium Is Partially Reversible

**Figure 2:**
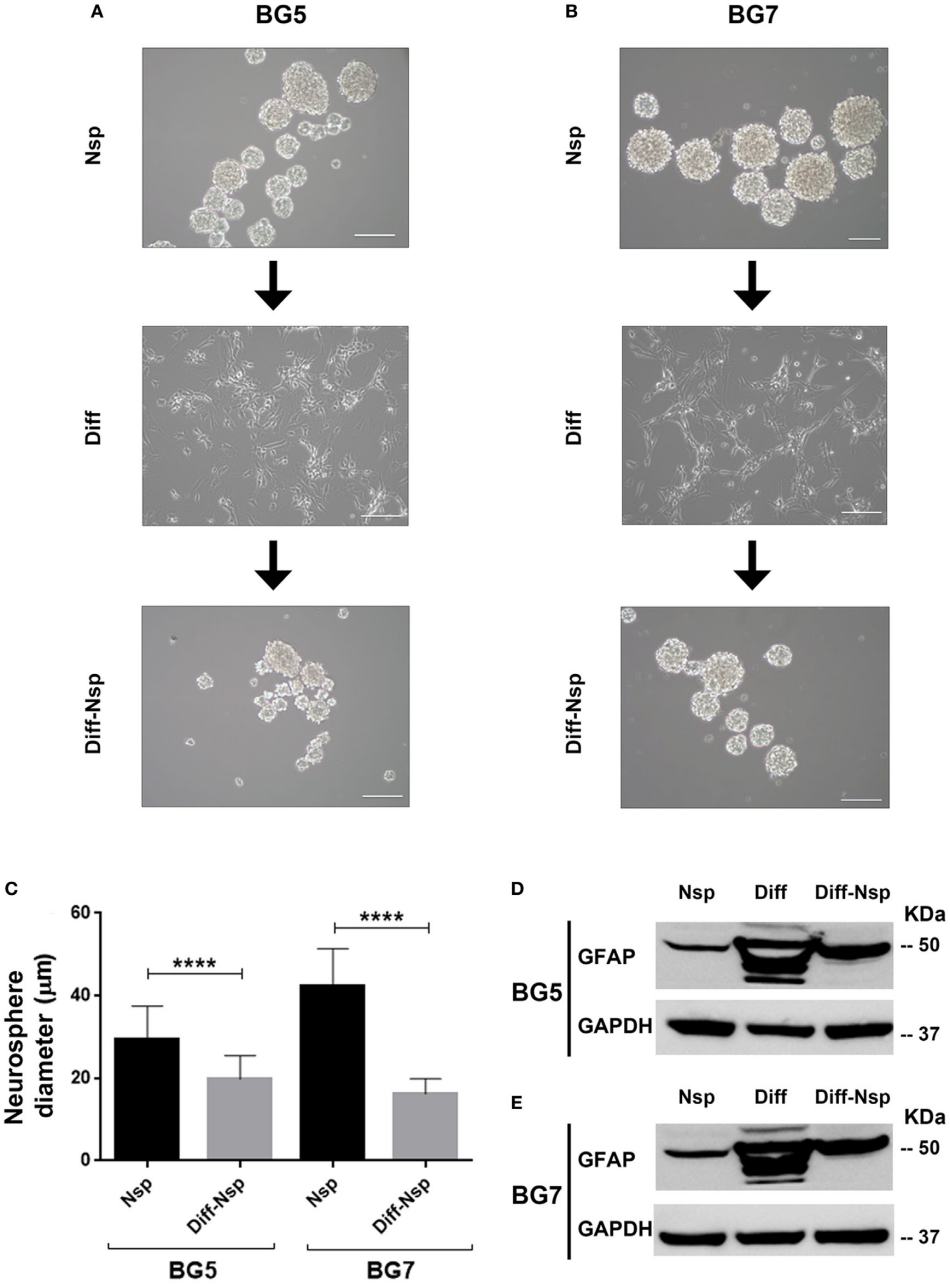
Differentiation and reversal of differentiation. Neurospheres (Nsp), differentiated to adherent cells (Diff) in 10% serum-containing medium and reverted back to a de-differentiated state (Diff-Nsp) after 14 days in stem-cell medium, **(A)** BG5 and **(B)** BG7 primary tumors. **(C)** Bar graph showing Nsp diameter in Nsp (black) and Diff-Nsp (gray) in BG5 and BG7 glioblastoma (GBM) cells. Data represent mean ± SD of *n* = 3 independent experiments per GBM patient-derived line. One-way ANOVA, Bonferroni’s multiple comparison test, ****P < 0.0001. Western blot showing glial acidic fibrillary protein and GAPDH in Nsp, differentiated, and de-differentiated Nsp **(D)** BG5 and **(E)** BG7 GBM cells.

To investigate whether GBM differentiation in serum-containing medium could be reversed, we “de-differentiated” the differentiated GG1 and GG9 cells (Figure S1 in Supplementary Material) as well as BG5 and BG7 GBM cells (Figure 2), propagated them for 14 days in stem cell medium and enumerated the size and number of the reaggregated neurospheres as a measure for self-renewal ability. In BG5 and BG7 the diameter of the “de-differentiated” neurospheres was reduced (Figures 2A–C, *P* < 0.0001), and limited dilution assay showed the number of neurospheres formed changed in GG1 and GG9 (Figures S1A–D in Supplementary Material, respectively, *P* < 0.0001). The differentiated cells highly expressed multilineage markers including GFAP (Figures 2D,E for BG5 and BG7), similar to GG1 and GG9 cells, where GFAP was subsequently upregulated as well as Olig2 (oligodendrocytes) and βIII tubulin (neurons) (Figure S1A in Supplementary Material for GG1; Figures S1B,E in Supplementary Material for GG9). Interestingly, in the “de-differentiated” cells GFAP expression levels were normalized in BG5 and BG7 cells (Figures 2D,E, respectively) as well as in GG9 cells (Figure S1B,E in Supplementary Material). Taken together, these data indicate that differentiation in serum-containing medium is partially reversible in short-term cultures.

### Differentiated GBM Cells Have Slower Proliferation Rates and PDT

**Figure 3:**
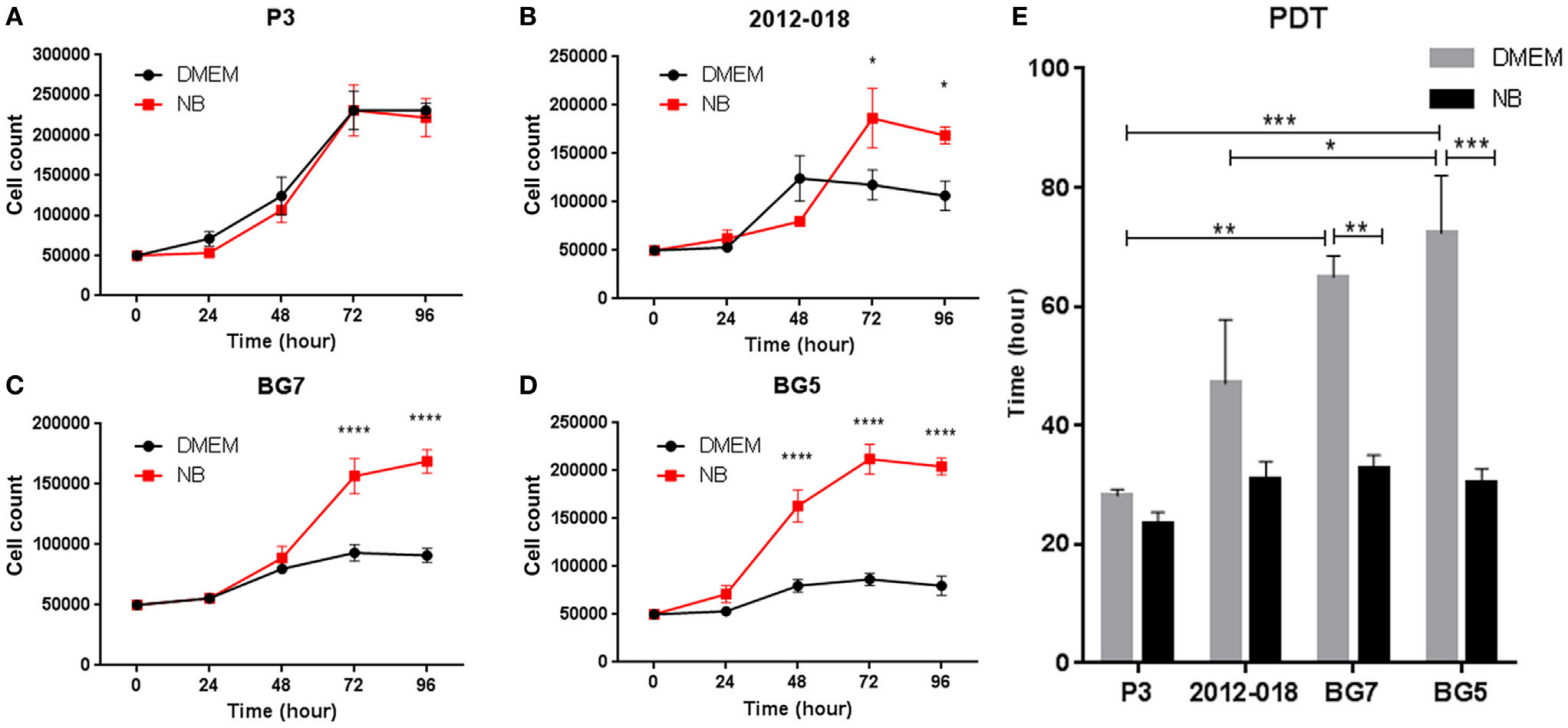
Differentiated glioblastoma (GBM) cells have slower proliferation rates and population doubling time (PDT). Growth curves showing number of **(A)** P3, **(B)** 2012-018, **(C)** BG7, and **(D)** BG5 GBM cells after 0, 24, 48, 72, and 96 h cultured in Dulbecco’s modified eagle medium (DMEM) (black) or NB (red). **(E)** PDT of GBM cells cultured in DMEM (gray) or NB (black). Data represent mean ± SEM of *n* = 3 independent experiments per GBM patient-derived cell line. Two-way ANOVA, Bonferroni’s multiple comparison test, *P < 0.05; **P < 0.01; ***P < 0.001; ****P < 0.0001.

We next investigated whether the changes in morphology and phenotype induced by the culture media might also be reflected in the cells’ growth potential. The culture medium had no effect on the growth of P3 cells (Figure 3A), whereas greater proliferation at 72 and 96 h (two-way ANOVA, *P* < 0.05, Figure 3B) was evident in 2012-018 GBM cells maintained in stem cell medium compared to those maintained in serum-containing medium. These differences in cell proliferation at 72 and 96 h were more pronounced for BG7 cells (two-way ANOVA, *P* < 0.0001, Figure 3C) and BG5 cells where robust cell proliferation was already evident after 48 h (two-way ANOVA, *P* < 0.0001, Figure 3D). The culture media had no effect on the PDT of P3 and 2012-018 GBM cells (*P* > 0.05, Figure 3E), but in BG7 and BG5 GBM cells, the time required to double their volume was significantly reduced when propagated in stem cell compared to serum-containing medium (two-way ANOVA, *P* < 0.01 for BG7 and *P* < 0.001 for BG5, Figure 3E). Taken together, these data indicate that GBM cells maintained in less differentiated states exhibit greater proliferative growth.

### Undifferentiated GBM Cells Are More Susceptible to NK Cell Lysis

**Figure 4:**
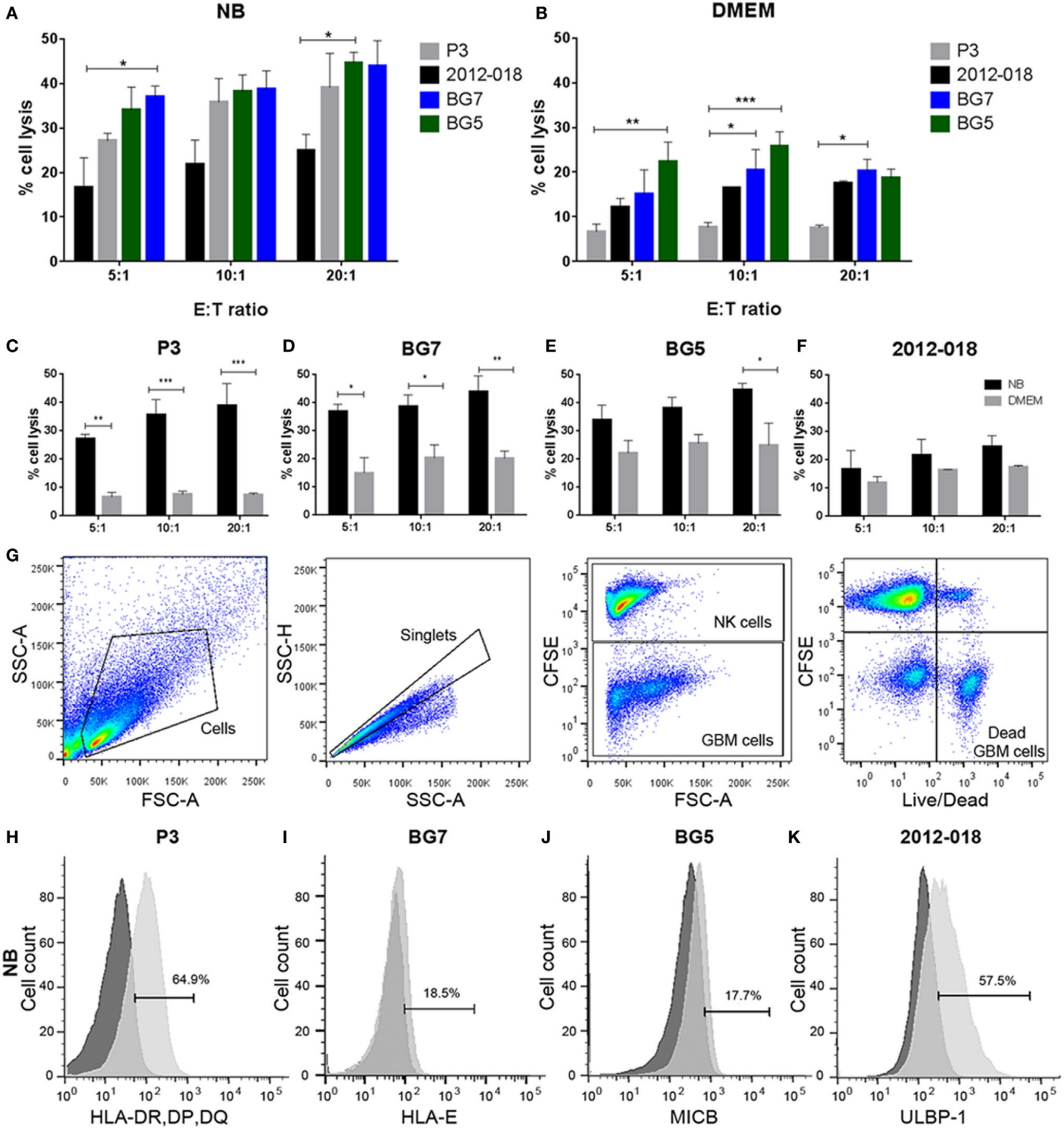

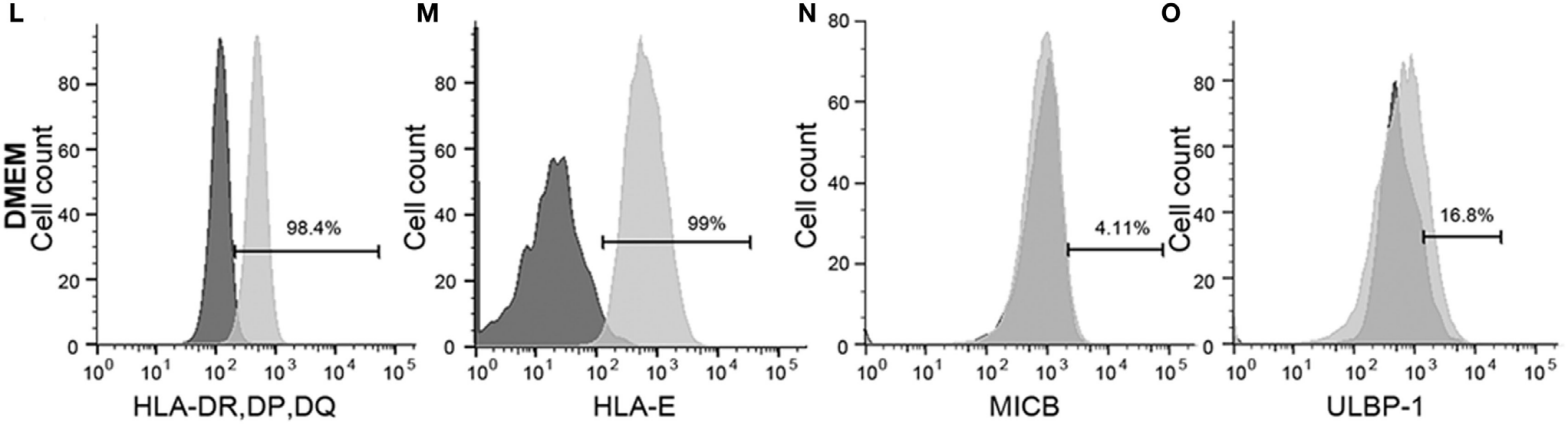
Undifferentiated glioblastoma (GBM) cells are more susceptible to natural killer (NK) cell lysis. Comparison of % lysis by NK cells of P3 (gray), 2012-018 (black), BG7 (blue), and BG5 (green) GBM cells cultured in **(A)** NB or **(B)** Dulbecco’s modified eagle medium (DMEM) media at E:T ratios 5:1, 10:1, and 20:1.% lysis by NK cells of **(C)** P3, **(D)** BG7, **(E)** BG5, and **(F)** 2012-018 GBM cells cultured in DMEM (gray) or NB (black) media at E:T ratios 5:1, 10:1, and 20:1. Data represent mean ± SEM of *n* = 3 independent experiments per GBM patient-derived cell line. Two-way ANOVA, Bonferroni’s multiple comparison test, *P < 0.05; **P < 0.01; ***P < 0.001. **(G)** Gating strategy for cytotoxicity analyses showing dot plots (from left to right): cells, single cells, separation of effector (NK cells), and target (GBM cells) cells, and live/dead segregation of effector and target cells. Histograms showing surface expression % of **(H)** human leukocyte antigen-DR, DP, DQ on P3, **(I)** HLA-E on BG7, **(J)** MICB on BG5, and **(K)** ULBP-1 on 2012-018 GBM cells cultured in NB and **(L–O)** in DMEM, respectively. Dark gray histograms—fluorescent minus one control and light gray histogram—expression of ligand.

We next investigated whether phenotypic changes induced by the culture media might affect the GBM cells’ responses to lysis by activated donor-derived NK cells. When maintained in stem cell medium most cells were equally sensitive to NK lysis, except for 2012-018 cells compared to BG7 and BG5 cells at E:T ratios 5:1 and 20:1, respectively (two-way ANOVA *P* < 0.05, Figure 4A). BG5 and BG7 cells propagated in DMEM were more sensitive than P3 cells to NK cell lysis (BG5 E:T ratios of 5:1 and 10:1, two-way ANOVA, *P* < 0.01 and *P* < 0.001, respectively, and BG7 cells E:T ratios 10:1 and 20:1, *P* < 0.05, Figure 4B). P3 and 2012-018 serum-cultured GBM cells were co-cultured with NK cells derived from donors 25, 35, and 59, while those maintained in stem cell medium were co-cultured with NK cells from donors 73, 74, and 75, Table 2. Additionally, BG5 cells maintained in both serum-containing and stem cell medium were co-cultured with NK cells derived from donors 25, 35, and 59, and BG7 with NK cells derived from donors 25, 35, and 75, Table 3. This allowed comparison across conditions, since it is established that the type and the number of receptor/ligand interactions are crucial for the potency of NK-mediated killing of target cells (44).

**Table 2:** Killer immunoglobulin-like receptors (KIR)-human leukocyte antigen (HLA) genotype table for P3 and 2012-018 with healthy donors used for cytotoxicity assays.

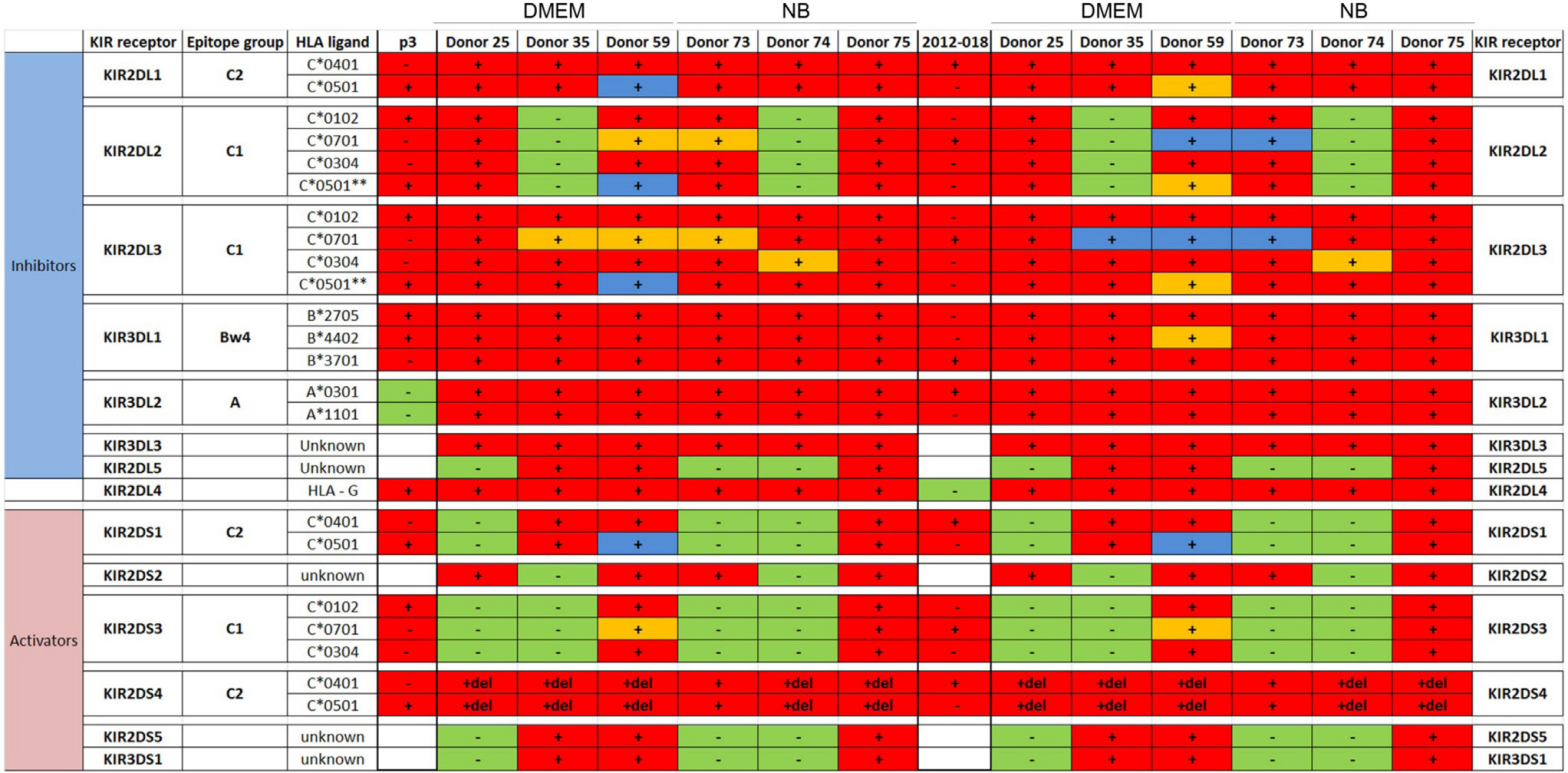

For natural killer (NK) cells: − and green, NK cells are negative for KIR; + and red, NK cells are positive for KIR; +del and red, NK cells have partial deletion KIR2DS4; +and blue, NK cells have been educated in the context of the cognate HLA, which is present in the glioblastoma (GBM); and +and orange, NK cells have been educated in the context of the cognate HLA, which is missing in the GBM, indicating a potential functional mismatch of inhibitory KIRs. For GBM: +, GBM are positive for the specific HLA ligand; −, GBM are negative for the HLA ligand. Red, GBMs are positive for at least one of the HLA ligands of the cognate KIR; green, GBMs are negative for all HLA ligands of the cognate KIR; and white, HLA ligand is unknown. **C2 epitope recognized by KIR2DL2/3.

**Table 3:** Killer immunoglobulin-like receptors (KIR)-human leukocyte antigen (HLA) genotype table for BG5 and BG7 with healthy donors used for cytotoxicity assays.

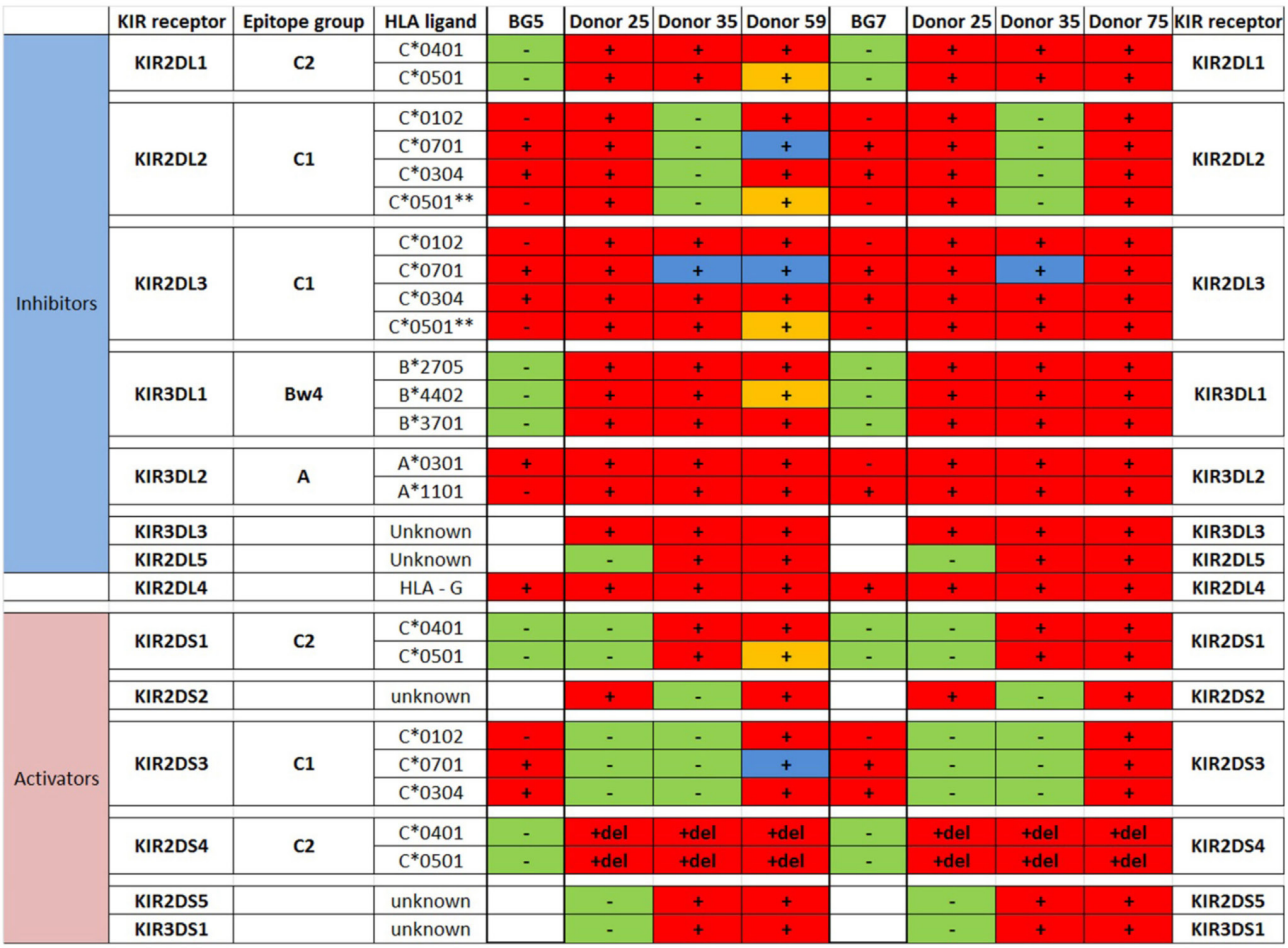

For NK cells: − and green, NK cells are negative for KIR; + and red, NK cells are positive for KIR; + del and red, NK cells have partial deletion KIR2DS4; + and blue, NK cells have been educated in the context of the cognate HLA, which is present in the glioblastoma (GBM); and + and orange, NK cells have been educated in the context of the cognate HLA, which is missing in the GBM, indicating a potential functional mismatch of inhibitory KIRs. For GBM: +, GBMs are positive for the specific HLA ligand; −, GBMs are negative for the HLA ligand. Red, GBMs are positive for at least one of the HLA ligands of the cognate KIR; green, GBMs are negative for all HLA ligands of the cognate KIR; and white, HLA ligand is unknown. **C2 epitope recognized by KIR2DL2/3.

To determine the contribution of KIR–HLA ligand interactions to the differential GBM killing efficacy in stem cell versus serum-containing medium, we genotyped the NK cells’ KIR receptors and their cognate HLA ligands on the GBM cells (Tables 2 and 3). Three inhibitory KIR–HLA ligand mismatch in donor 35 and 59’s KIR2DL2 and KIR2DL3 with HLA-C*07:01 ligands (P3) and four inhibitory KIR–HLA ligand mismatch with ligands HLA- C*05:01 and Bw4 B*4402 (for KIR2DL1, KIR2DL2, KIR2DL3 for C*05:01 and KIR3DL1 for B*4402 in 2012-018 cells) were present under serum-culture conditions (Table 2), diminishing NK cell inhibition through the “missing self” mechanism, thus promoting potency against these cells (Figures 4B,C,F). However, donor 59’s KIR2DL1, KIR2DL2, and KIR2DL3 licensed NK cells encountering HLA-group C1 ligands C*05:01 (present in P3 cells) or licensed NK cells from donors 59 and 35 encountering C*07:01 alleles also present in 2012-018 cells might have “calibrated” the killing potency against the GBM cells in serum-containing medium (Table 2; Figures 4B,C,F).

In contrast, three inhibitory KIR–HLA ligand mismatch for KIR2DL2 and KIR2DL3 against HLA-C*07:01 were present in P3 cells co-cultured in stem cell medium (Table 2; Figures 4A,C). Diminished killing of 2012-018 cells in stem cell medium may have been due to licensed KIR2DL2 and KIR2DL3 NK cells to cognate HLA-C*07:01 alleles that were also present in the GBM cells (Table 2; Figure 4A), thus calibrating these NK cells’ potency and explaining why there was no difference in responses of these cells to NK cell lysis between the two culture media (Table 2; Figure 4F).

In BG7 GBM cells that were highly sensitive for NK cell-mediated lysis (Figures 4A,B,D), the inhibitory signals of intermediate strength from licensed KIR2DL3 against cognate ligand HLA-C*07:01 (also present in BG7 cells) might have been overridden by further lack of KIR2DL2 in these donors’ NK cells that also possessed several non-licensed, potentially hyporesponsive KIR subsets (Table 3). No significant difference in cytolysis of BG5 cells was noted in stem cell or serum-containing medium, except at high E:T ratio 20:1 (Figure 4E). Despite HLA ligand mismatch to strongly inhibitory KIR2DL1, as well as KIR2DL2, KIR2DL3, and KIR3DL1 of donor 59’s NK cells thereby permitting penetrance of activating signals, HLA-C*07:01 licensed KIR2DL2 and KIR2DL3 NK cells from donors 35 and 59 encountering HLA-C*07:01 ligands present in the BG5 cells (Table 3), may have offset the activation signals. Taken together, KIR–HLA ligand mismatch between the licensed NK cells, particularly of strongly inhibitory KIR2DL1, and GBM cells’ cognate HLA ligands at the genomic level contributed to susceptibility for NK cell-mediated lysis.

Expression of classical as well as non-classical HLA ligands for inhibitory KIRs, including HLA-G were reduced in GBM cells maintained in stem cell medium (Table 1; Figures 4H,I,L,M) when these GBM cells were also more susceptible to NK cells. Greater GBM cell fractions from all tumors examined also expressed ICAM-1 under stem cell conditions but attenuated expression in serum-containing medium (Table 1).

### Serum-Cultured GBM Cells Less Sensitive for NK-Mediated Cell Lysis Downregulate Activating and Upregulate Inhibitory Ligands for NK Cell Receptors

**Figure 5:**
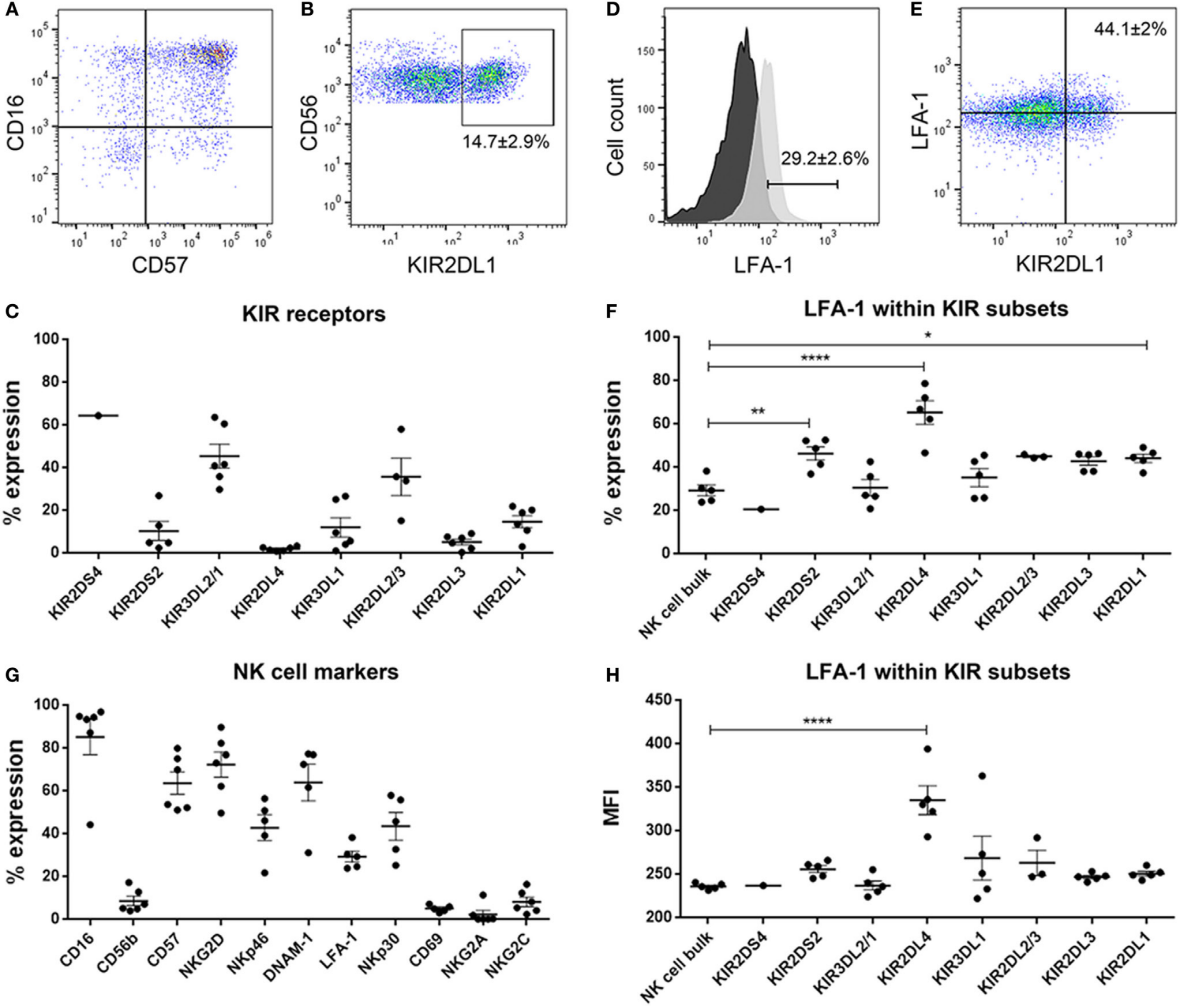
Dot plots showing **(A)** CD16 versus CD57 and **(B)** CD56 versus KIR2DL1 natural killer (NK) cells. % expression of **(C)** killer immunoglobulin-like receptors (KIR) receptors on donor-derived-NK cells. **(D)** Histograms showing surface expression % of LFA-1 in NK cell bulk. Black histogram—fluorescent minus one control and light gray histogram—expression of LFA-1 in sample. **(E)** Dot plot showing LFA-1 versus KIR2DL1 in representative NK cells. % expression of **(F)** LFA-1 within the KIR subsets and **(G)** NK cell markers on donor-derived-NK cells. **(H)** MFI of LFA-1 within the KIR subsets. Data represent mean ± SEM of *n* = 6 donor-derived NK cells. One-way ANOVA, Bonferroni’s multiple comparison test, *P < 0.05; **P < 0.01; ****P < 0.0001.

As the findings above still do not unequivocally establish interactions within KIR2DL1 subsets mismatched for cognate HLA ligand as the minimal requirement for NK cell lysis of GBM cells, we hypothesized that culture conditions might further modulate GBM susceptibility to NK cell killing by changing surface expression of inhibitory and stimulatory ligands recognized by NK cells’ NCRs. P3 GBM cells cultured in stem cell medium succumbed more readily to NK cell lysis compared to when cultured in serum-containing medium at all E:T ratios (two-way ANOVA, *P* < 0.01, 5:1, and *P* < 0.001 for both 10:1 and 20:1, Figure 4C). This reduced susceptibility of P3 cells to NK cell lysis corresponded to attenuated proportion of cells expressing ULBP-1 and MICB; B7-H6 and CD112 representing important ligands for the NK cell activating receptors NKG2D; NKp30 and DNAM-1, respectively (Table 1), which were correspondingly highly expressed on the surface of the CD57^+^CD16^+^ donor NK cells (Figures 5A,G). Classical HLA–ABC as well as non-classical HLA-DR, DP, and DQ (Figure 4L) were highly expressed in P3 cells propagated in serum-containing medium, Table 1.

Likewise, BG7 cells propagated in stem cell medium were more susceptible to NK cell lysis at all E:T ratios (two-way ANOVA, *P* < 0.05, Figure 4D) when all the activating ligands (ULBPs and MICs) for NKG2D and CD112 for DNAM-1 receptors were highly expressed and the inhibitory (both classical and non-classical HLA ligands) were concomitantly downregulated, Table 1; Figures 4M,I. BG5 cells were receptive to NK lysis only at E:T ratio 20:1 (two-way ANOVA, *P* < 0.05, Figure 4E). The ULBP-2/5/6, ULBP-3, MICA, and MICB (Table 1; Figures 4J,N) ligands for activating NKG2D as well as B7-H6 and CD112 ligands for NKp30 and DNAM-1 receptors were upregulated, while expression of inhibitory ligands HLA–ABC, HLA-E, and HLA-G were largely unchanged between the culture media, Table 1, potentially explaining the moderate NK cell efficacy at 20:1 E:T ratio. In contrast, non-classical HLA-DR, DP, and DQ were highly expressed, Table 1.

In contrast, 2012-018 cells were most resistant to NK cell lysis denoted by the low killing efficiency regardless of culture media (Figure 4F). Although ULBP-1 (Figures 4K,O), ULBP-3, and ULBP-2/5/6 stress ligands recognized by NKG2D and B7-H6, CD112 ligand recognized by NKp30, DNAM-1 activating receptors, respectively, were diminished in the serum-cultured 2012-018 GBM cells, inhibitory classical HLA–ABC ligands were markedly upregulated in 2012-018 GBM cells when cultured in serum-containing medium (Table 1). Since ligands for activating receptors were equally diminished as ligands for inhibitory receptors were upregulated in the 2012-018 cells, this might explain their reduced sensitivity for NK cell-mediated lysis and why media had no significant effect.

The donor NK cells had predominantly mature phenotype of CD57^+^CD16^+^ (Figures 5A,G) and robustly expressed surface KIR, verified by flow cytometry where 14.7 ± 2.9% of the NK cells were KIR2DL1 (Figures 5B,C) and 12 ± 4.5% were KIR3DL1 positive; 45.36 ± 5.6 and 35.7 ± 8.8% were KIR3DL2 and KIR2DL2, respectively, Figure 5C. KIR2DS2, KIR2DL4, and KIR2DL3 were barely expressed (Figure 5C). It is established that in order for NK cells to kill, they must first engage their targets to receive activation signals through β2 integrin receptor LFA-1 interaction with its ligand ICAM-1, and that this interaction is required for NK cell-mediated lysis (45, 46). Thus, we established that approximately 30% of the bulk NK cell population was potentially highly cytotoxic based on LFA-1 expression (Figures 5D,F) and proceeded to investigate whether these corresponded to particular KIR clones in order to elucidate which KIR receptors contributed most killing potency to the KIR–HLA ligand mismatch. For this we examined the KIR subsets expressing LFA-1 and their interaction with GBM cells that differentially expressed ICAM-1 within the culture conditions (Figure 5F). Greater fractions of KIR2DL1 NK cell clones that were KIR–HLA ligand mismatched against ICAM-1 expressing GBM cells expressed LFA-1 compared to bulk NK cells (*P* < 0.05, Figures 5E,F). KIR2DL4 and KIR2DS2 NK cell subsets also expressed LFA-1 (*P* < 0.0001 and *P* < 0.01, respectively, Figures 5F,H), despite negligible surface expression of KIR2DL4 (Figure 5C). Taken together, reduced class I-HLA ligands, together with increased ICAM-1/LFA-1 interactions within KIR2DL1 receptor might tip the threshold balance toward increased activation, effector target conjugation, and increased killing of GBM cells maintained in stem cell media.

## Discussion

Immunotherapy has emerged as a successful treatment of choice for several cancers. However, this modality has yet to reap benefit for patients whose tumors are characterized by great molecular and cellular heterogeneity, as well as immunological tolerance that render them refractory to T- and dendritic cell vaccine-based immunotherapy. These approaches require high mutational load generating immunogenic neoantigens that must be subsequently presented appropriately to professional antigen presenting cells with robust co-stimulatory signals (47). These conditions are hard to establish in the immunosuppressive brain tumor microenvironment (9, 48). Thus, NK cells hold great promise as immunotherapy effectors against GBM, since they broadly recognize transformed and stressed cells in a manner that is unrestricted by HLA bound tumor neoantigens, while sparing healthy cells. This feature is particularly important for counteracting GBM’s high molecular and cellular heterogeneity (11), where antigen loss cell variants mediate immunological escape and resistance to immunotherapy (6, 49, 50). However, it is currently unclear in pre-clinical studies evaluating potential of NK cell therapy how routinely used culture media affect primary GBM cell responses to NK-mediated cytotoxicity. Several studies claimed that GBM cells were refractory from NK lysis due to high expression of inhibitory class I HLA ligands, expression of HLA-E ligand for inhibitory CD94/NKG2A receptor, as well as poor surface expression of ligands for activating NKG2D receptor (34, 35).

In this present study, we investigated paired analyses, six primary GBM cells cultured in stem cell versus serum-containing medium to elucidate differences in morphology, phenotype, growth, and responses to NK cytotoxicity from six random donors. We report that the GBM cells maintained in stem-cell medium displayed phenotypes consistent with immature glioma stem-like cells, characterized by growth as neurospheres, increased proliferation and elevated expression of nestin, but diminished lineage differentiation markers. Upon transfer and propagation in serum-containing medium, these cells became adherent and switched to epithelioid mesenchymal morphology, consistent with differentiation (36, 38) (which nevertheless could be reversed in short-term 14 day assay), proliferation was attenuated, as expression of nestin, while GFAP and A2B5 levels were upregulated. Expression of putative stem cell markers CD15 and CD133 (Prominin-1) varied with tumor type, independent of medium conditions. The use of CD133 to identify GBM stem cells (GSCs) has remained contraversial (51) due to unreliability of distribution, as CD133 is also expressed on long-term established GBM cell lines and thus, calls into question its specificity as stem cell marker and its prognostic validity in patients. Through limiting dilution experiments, CD133^−^ glioma cells were equally able to self-renew and retain tumorigenic potential, a feature supposedly reserved for putative, rare GSCs (39). In the current study, both CD133 and CD15 stem cell markers did not correlate with GSC phenotype, but instead exhibited tumor dependent expression regardless of culture media condition. A2B5 that was also reported to be a marker of less differentiated cancer cells (52), however, in our hands it was consistently highly expressed on the differentiated, adherent cells propagated in serum-containing medium. Since this antibody identifies a cocktail of ganglioside epitopes, it may rather identify cells with increased propensity for adhesion. A2B5^+^ cells and indeed, exogenously added gangliosides promote GBM invasion, likely due to their promotion of adhesion to the basement membrane (53). Thus, changes in expression density of nestin and GFAP intermediate filaments appeared to be most reliable indication for loss of stemness in differentiating serum-containing medium. As the differentiated cells could be “de-differentiated” in the short-term to re-acquire self-renewal, neurosphere forming properties, it may indicate the great plasticity possible in GBM cells *in situ*, under the influence of various microenvironmental factors, such as hypoxia and inflammatory cytokines (54, 55). Our findings corroborate, to a large extent, previous studies that demonstrated similar phenotypic changes in primary GBM cells cultured in stem cell *versus* serum-containing medium (36, 56, 57). These authors further demonstrated that stem cell cultured GBM cells bore remarkable similarity to normal neural stem cells, due to ability to form neurospheres *in vitro* and potential for self-renewal. Additionally, it is established that serum-cultured cells accumulate mutations and differ substantially from the parental tumor (37). Collectively, the prior findings brought into the question the relevance of standard cell lines for studying the biology of human cancers. This conclusion garnered the impetus for undertaking the current study to investigate not only biological behavior of primary GBM cells under these conditions, but principally, their responses to NK cytotoxicity.

The major aim of this work was to determine whether GBM susceptibility to NK cell-mediated lysis was different for cells maintained in stem cell-compared to serum-containing medium and elucidate the potential mechanisms underlying this. We found robustly augmented NK cell cytotoxicity against stem cell-like GBM cells compared to differentiated cells maintained in serum-containing medium. Our findings partially corroborate earlier work (56, 57) demonstrating susceptibility of GBM stem-like cells to lysis by both allogeneic and autologous NK cells activated with IL-2 or IL-15, but not resting NK cells. Intriguingly, it was reported that co-culture of tumor cells with IL-2-activated NK cell induced so-called “split-anergy” where the cells downregulated CD16, were less cytotoxic but continued to secrete IFNγ that promoted tumor differentiation, elevated class I HLA, possibly mediating immunological escape *in vivo* (58). Indeed in support of these findings, we previously demonstrated that bulk NK cells activated in IL-2 *in vitro* (9), as well as tumor-infiltrating NK cells in GBM biopsies *in situ* exhibited these phenomena (42), whereas highly potent KIR2DS2 subsets retained high CD16 expression upon encounter with undifferentiated GBM targets, secreted IFNγ, and were highly potent.

Notwithstanding the complexity posed by multiple receptor–ligand interactions between the NK cells and GBM target cells, and the relatively small sample sizes, we found multiple differences in the expression of ligands for activation and inhibitory NK cell receptors. All cells cultured under differentiating, serum conditions were less sensitive to NK cell lysis and expressed lower levels of stress ligands for NKG2D, B7-H6, and CD112 recognized by NKp30 and DNAM-1 NCRs, respectively. We previously demonstrated that blocking the NKG2D receptor on NK cells abolished cytotoxicity against GBM by 50% (42), underscoring the significance of the findings reported herein. In contrast, we showed that differentiated GBM cells upregulated classical as well as non-classical HLA-DR, DP, and DQ ligands, including HLA-G. The latter serves as ligand for KIR2DL4 (59–61), where surface expression in melanoma cells was demonstrated to inhibit NK lysis (62). In long-term established GBM cell lines, HLA-G was reported expressed on few tumor cells, where inhibitory signals were directed against CD8^+^ and CD4^+^ T cells but not NK cells (63). In contrast, we found HLA-G to be highly expressed in GBM primary cultures regardless of culture conditions, however, the cognate KIR2DL4 receptor was expressed on few donor NK cells, which densely co-expressed LFA-1. Thus, role of these few KIR2DL4/LFA-1^+^ NK cell subsets in the context of diminished ICAM-1 but abundant HLA-G expression in differentiated GBM cells is uncertain. Previous studies (57) also reported reduced expression of class I HLA ligands in stem-cell-like GBM cells and concluded that increased susceptibility to NK cells under these conditions was mediated through these low, non-protective levels of class I HLA ligands. However, antibody blockade of class I HLA molecules on serum-cultured GBM cells did not relinquish resistance to NK lysis to levels of their stem-cell cultured counterparts (57), suggesting that additional resistance mechanisms against NK lysis occur in serum-cultured GBM cells.

In our study, we examined the impact of inhibitory KIR–HLA ligand interactions at the genomic level and found that NK cell potency against GBM was influenced by the presence of licensed KIR encountering cognate HLA ligands in the patients’ tumor, thereby calibrating their efficacy. Inhibitory KIR–HLA ligand mismatch within the licensed KIR could potentially offset strongly inhibited NK cells. It is difficult to unequivocally and empirically show the minimal requirements for NK cell cytotoxicity, especially deciphering the individual contribution of inhibitory KIR receptors for the differentially expressed class I HLA ligands. This is because the KIR genes on chromosome 19q13 are inherited independently from their HLA ligands encoded on chromosome 6 (16–18) resulting in multiple, exquisitely overlapping receptor–ligand interactions. Furthermore, KIR surface expression is stochastic, although generally, the number of single KIR^+^ clones correlate with the presence of the educating self-ligand (64). KIR polymorphisms have been observed in all inhibitory KIRs genes and offer a third level of complexity, where, for example, in KIR2DL1, upto 25 alleles have been reported, each with different strength of inhibiting NK cell lysis and duration of expression on the surface after interaction with ligands (65, 66). This exquisite complexity makes KIR cloning from primary cells a formidable challenge, steering a preference to work with bulk, un-manipulated primary NK cell cultures. Furthermore, due to unrestricted availability of fresh genoptyped primary samples, we could not use cloning to derive the KIR subsets individually contributing to GBM cytotoxicity. However, since expression of the β2 integrin receptor (CD11/CD18; LFA-1) in NK cells is crucial for NK-target conjugate formation at the immunological synapse, we investigated the LFA-1^+^/KIR^+^ subsets’ potential interaction with ICAM-1-expressing GBM targets. LFA-1/ICAM-1 interaction was previously demonstrated to induce inside-out signaling and a conformational change in β2 integrin, preferentially transmitting potent activation and polarization of cytolytic granules toward the targets (45, 46, 67). Since (1) NK cell potency is tuned by the presence of licensed KIR subsets encountering (or not) cognate HLA ligands on the target cells (17, 21, 66), (2) no particular marker exists to distinguish licensed from un-licensed NK cells, and (3) LFA-1/ICAM-1 interactions are required for the NK-target conjugation and potent killing, we investigated these interactions within the licensed KIR subsets involved in KIR–HLA ligand mismatch in order to evaluate the individual cytotoxicity contribution of the single KIRs. 44% of KIR2DL1 subsets expressed LFA-1 and were receptor–ligand mismatched against undifferentiated and susceptible ICAM-1 expressing GBM cells propagated in stem cell medium, potentially implicating KIR2DL1/HLA-C2 receptor–ligand interactions in the efficacy. As the strength of inhibitory interaction determines the licensed NK cells’ potency against encountered target cells lacking the appropriate HLA ligands (17, 21, 68), it is noteworthy that it was higher fractions of KIR2DL1 subsets that expressed LFA-1 and that were in KIR–HLA ligand mismatch with ICAM-1 expressing susceptible GBM cells. Surprisingly, KIR2DL2 and KIR2DL3 subsets licensed on HLA-C1/C2 ligands with intermediate and moderate potency, respectively, did not express LFA-1 above that of bulk NK cells. This can be explained by greater heterogeneity in HLA-C1/C2 licensing within these subsets among the donors, hence their greater heterogeneity in LFA-1 expression and intermediate/moderate cytotoxicity against ICAM-1 expressing GBM targets. Greater fractions of KIR2DS2 subsets expressed LFA-1, supporting our previous report that they potently kill undifferentiated GBM cells compared to bulk NK cells (42). Nevertheless, it may have been expected that greater fractions of bulk cells might express LFA-1. The signal from FITC-conjugated CD11/CD18 mAb 24 clone was not as bright as could be achieved with the other fluorochromes, but we chose to have the stochastically expressed KIRs, in bright and well proven antibodies and fluorochromes that could also be related to our previous publications, since specificity may be debated (69) due to the high homology between the KIRs. However, in this regard focusing on number of cells likely to form immunological synapse, polarize cytolytic granules, and potently kill targets is more relevant than the density of receptors on the surface (represented by mean fluorescence intensity). It is pertinent, since we did not examine LFA-1 in the context of open/active conformation change within the immune synapse relative to expression outside the synapse (70). One study (70) showed that approximately 40% bulk resting NK cells expressed LFA-1 in open conformation, corroborating our findings. We also examined LFA-1 expression in resting NK cells to avoid IL2 stimulation that might influence the fraction of expressed KIRs due to NK culture condition. Indeed, Enqvist et al. (70) found increased LFA-1 within KIRs in IL2 stimulated than bulk resting NK cells and could be confounded by culture effects. They also examined efficacy in ICAM-1 overexpressed cells, a measure that can remove physiological subtility in their data. We wanted to relate the level of KIR and LFA-1 expression to the level of education and potency against GBM targets.

Nevertheless, it is well established that KIR receptor–ligand mismatch mediates potent graft-versus-leukemia effect, improving the outcome of patients receiving haploidentical hematopoietic stem cell transplantation (24, 71), as well as play important roles in the cytolysis of solid tumor cells *in vitro* (42). Our study fills a knowledge gap in the previous studies conducted on this topic by demonstrating clearly that inhibitory KIR–HLA ligand interactions contribute to GBM susceptibility to NK cell lysis, particularly when examined within KIR subsets in LFA-1/ICAM-1 interactions and should be considered simultaneously with effects of stress ligands on NCRs. We further support the findings in the literature regarding importance of NKG2D, NKp30, and DNAM-1 activating receptor ligation to cognate ligands in NK cell-mediated GBM lysis. Based on these findings we concluded that the culture media altered GBM responses to NK cytotoxicity partially mediated through modulating the threshold balance of stress-induced ligands, NK cell activating, as well as inhibitory HLA ligands. The development of preclinical immunotherapy strategies against GBM should not use cells propagated in serum-containing media to avoid misinterpretation of potential therapeutic responses.

## Ethics Statement

Ethical approval for collection of tissue and blood samples was obtained from the regional committee of western Norway (REKvest 2013/720/) and The Norwegian Data Protection Agency. Ethical approval was also obtained (REKvest 2014/588) to collect blood from healthy blood donors from the Blood Bank and Transfusion Unit, Haukeland University Hospital. Patients and donors gave their informed consent to specimen collection for research in accordance with the Declaration of Helsinki.

## Author Contributions

MC: conceived study, designed research, acquired funding, and wrote the manuscript. AN: designed and conducted experiments, analyzed data, and revised manuscript. HH: performed experiments, analyzed data, and revised manuscript. MR: performed experiments, analyzed data, and revised manuscript. JJ: provided materials, conducted experiments, and revised manuscript.

## Conflict of Interest Statement

The authors declare that the research was conducted in the absence of any commercial or financial relationships that could be construed as a potential conflict of interest.

## Supplementary Material

Figure S1 Differentiation and reversal of differentiation. Left panel: primary glioblastoma (GBM), neurospheres (Nsp), differentiated (Diff) to adherent cells in 10% serum-containing and de-differentiated neurospheres (Diff-Nsp) in the stem cell medium; and right panel: immunofluorescence staining of the tri-lineage differentiation markers—glial acidic fibrillary protein (GFAP) (astrocytes), Olig2 (Oligodendrocytes), and βΙΙΙ tubulin (neurons) in **(A)** GG1 and **(B)** GG9 primary GBM lines. Number of original neuropsheres (black) versus de-differentiated neurospheres (gray) formed in **(C)** GG1 and **(D)** GG9 GBM cells. Data represent mean ± SD of *n* = 3 independent experiments. Two-way ANOVA, Bonferroni’s multiple comparison test, ****P* < 0.001; *****P* < 0.0001. **(E)** Western blot of GFAP and β-actin in neurospheres, differentiated, and de-differentiated GG9 GBM cells.
