# Supplementary figures and images for: Glioblastoma Stem-Like Cells Are More Susceptible Than Differentiated Cells to Natural Killer Cell Lysis Mediated Through Killer Immunoglobulin-Like Receptors–Human Leukocyte Antigen Ligand Mismatch and Activation Receptor–Ligand Interactions
Source: Front Immunol. 2018 Jun 18;9:1345. doi: 10.3389/fimmu.2018.01345 (PMC6015895; doi:10.3389/fimmu.2018.01345)

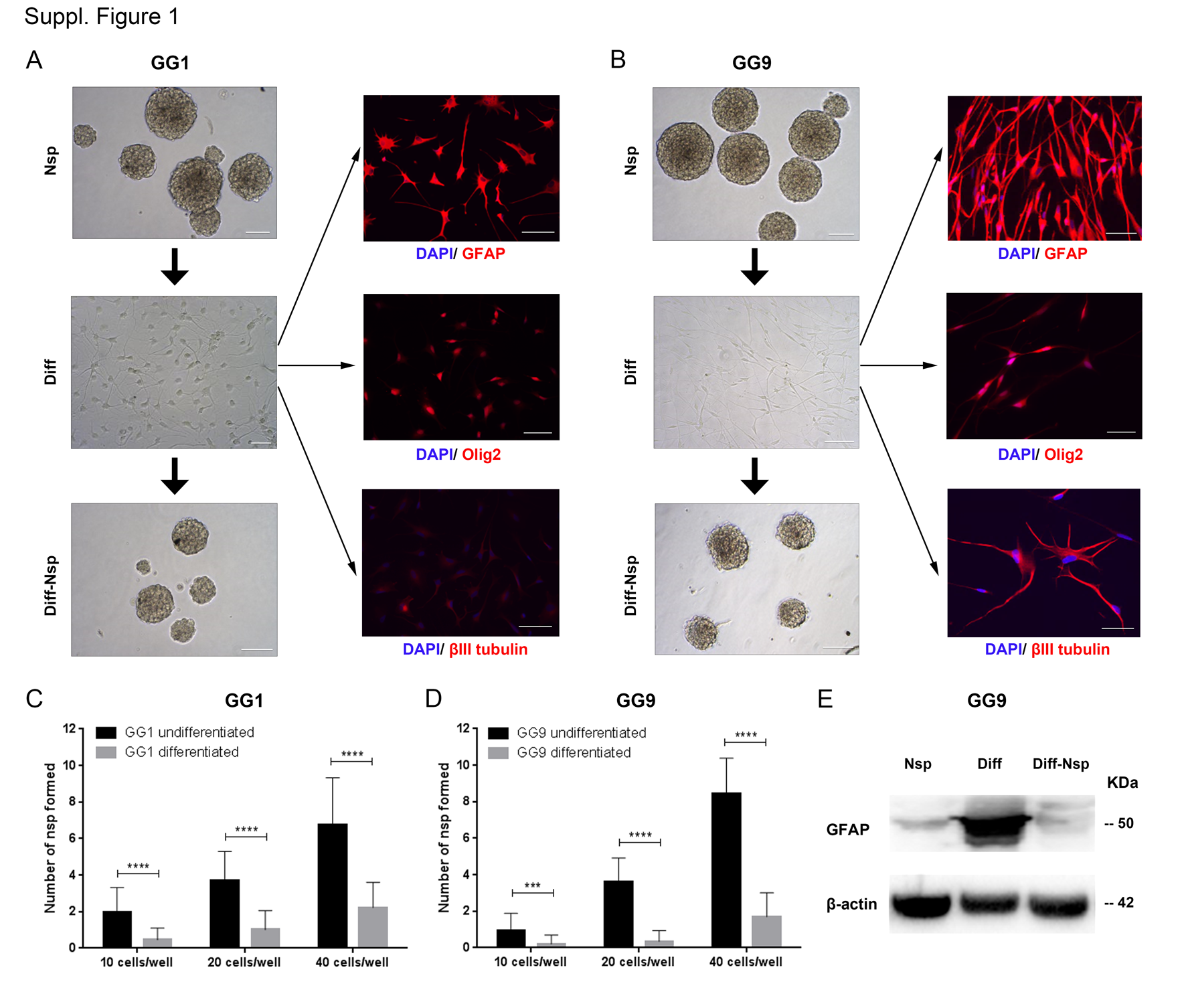

Supplement: Figure S1 — Differentiation and reversal of differentiation. Left panel: primary glioblastoma (GBM), neurospheres (Nsp), differentiated (Diff) to adherent cells in 10% serum-containing and de-differentiated neurospheres (Diff-Nsp) in the stem cell medium; and right panel: immunofluorescence staining of the tri-lineage differentiation markers—glial acidic fibrillary protein (GFAP) (astrocytes), Olig2 (Oligodendrocytes), and βΙΙΙ tubulin (neurons) in (A) GG1 and (B) GG9 primary GBM lines. Number of original neuropsheres (black) versus de-differentiated neurospheres (gray) formed in (C) GG1 and (D) GG9 GBM cells. Data represent mean ± SD of n = 3 independent experiments. Two-way ANOVA, Bonferroni’s multiple comparison test, ***P < 0.001; ****P < 0.0001. (E) Western blot of GFAP and β-actin in neurospheres, differentiated, and de-differentiated GG9 GBM cells. [file Image_1.tif]
