# Supplementary material for: Glioblastoma Stem-Like Cells Are More Susceptible Than Differentiated Cells to Natural Killer Cell Lysis Mediated Through Killer Immunoglobulin-Like Receptors–Human Leukocyte Antigen Ligand Mismatch and Activation Receptor–Ligand Interactions
Source: Front Immunol. 2018 Jun 18;9:1345. doi: 10.3389/fimmu.2018.01345 (PMC6015895; doi:10.3389/fimmu.2018.01345)
Supplement: Supplementary file 2 [file Table_1.docx]

**Supplementary Table I. List of antibodies used in flow cytometry, western blot and immunofluorescence**

| Antibody | Fluorochrome | Isotype and clone | Catalogue number | Supplier |
| --- | --- | --- | --- | --- |
| nestin | PerCP-Cy5.5 | IgG1, 25/NESTIN | 561231 | BD Biosciences |
| CD15 | PE-Cy7 | IgM, HI98 | 560827 | BD Biosciences |
| HLA-ABC | APC | IgG1, G46-2,6 | 562006 | BD Biosciences |
| vimentin | PE | IgG1, RV202 | 562337 | BD Biosciences |
| HLA-DR, DP, DQ | FITC | IgG2a, Tu39 | 562008 | BD Biosciences |
| MICB | APC | IgG2b, 236511 | FAB1599A | R&D Systems |
| MICA | APC | IgG2B, 159227 | FAB1300A | R&D Systems |
| ULBP-1 | # | IgG2a, 170818 | MAB1380 | R&D Systems |
| ULBP-2/5/6 | # | IgG2a, 165903 | MAB1298 | R&D Systems |
| ULBP-3 | # | IgG2a, 166510 | MAB1517 | R&D Systems |
| A2B5 | PE | IgM, 105-HB29 | 130-093- 581 | Miltenyi Biotec |
| CD133 | APC | IgG1, AC133 | 130-090- 826 | Miltenyi Biotec |
| GFAP | FITC | IgG1, GA5 | 53-9892 | eBioscience |
| HLA-E | PE | IgG1, 3D12HLA-E | 12-9953 | eBioscience |
| CD31 | PE-Cy7 | IgG1, WM-59 | 25-0319 | eBioscience |
| HLA-G | PE | IgG2a, 87G | 12-9957 | eBioscience |
| HLA-A3 | ¤ | IgM, 4i153 | Ab31572 | Abcam |
| HLA-Bw4 | ¤ | IgM, 4i121 | Ab34049 | Abcam |
| ¤ F(ab’)2 | FITC | Rabbit anti-mouse IgM | 61-6811 | Invitrogen |
| # F(ab’)2 | Pac. Orange | Goat anti-mouse IgG | P31585 | Invitrogen |
| CD54 | FITC | IgG1, HA56 | 353107 | Biolegend |
| CD112 | PerCP-Cy5.5 | IgG1, TX31 | 337416 | Biolegend |
| B7-H6 | PE | IgG1, 875001 | FAB7144P | R&D Systems |
| LFA-1 | FITC | IgG1, m24 | 363416 | Biolegend |
| CD226 | PE | IgG1, 11A8 | 338305 | Biolegend |
| CD56 | V450 | IgG1, B159 | 560360 | BD Bioscience |
| CD3 | V500 | IgG1, SP34-2 | 560770 | BD Bioscience |
| CD16 | FITC | IgG1, 3G8 | 555406 | BD Bioscience |
| CD69 | PE | IgG1, FN50 | 555531 | BD Bioscience |
| NKG2D | PE-Cy7 | IgG1, 1D11 | 562365 | BD Bioscience |
| NKp46 | APC | IgG1, 9-E2 | 558051 | BD Bioscience |
| NKG2A | Alexa Fluor® 700 | IgG2a, 131411 | FAB1059N | R&D System |
| CD57 | PE-Cy5 | IgM, NK-1 | Ab25445 | Abcam (UK) |
| KIR2DL4 | APC | IgG2a, 181703 | FAB2238A | R&D System |
| KIR2DL1 | Alexa Fluor® 700 | IgG1, 143211 | FAB1844N | R&D System |
| KIR2DL3 | APC | IgG2a, 180701 | FAB2014A | R&D System |
| KIR2DS4 | PE | IgG2a, 179315 | MAB1847 | R&D System |
| KIR3DL1 | Alexa Fluor® 700 | IgG1, DX9 | 312712 | Biolegend |
| KIR2DL2/3 | PE | IgG2a, DX27 | 130-092-618 | Miltenyi Biotec |
| KIR3DL1/2 | PE | IgG1, 5.133 | 130-095-205 | Miltenyi Biotec |
| KIR2DS2 | * | Rabbit polyclonal | ab175486 | Abcam |
| * F(ab’)2 | Qdot585 | Goat anti-rabbit IgG | Q-11411MP | Invitrogen |
| Live/Dead  Fixable Near-IR | APC-Cy7 | - | L10119 | Invitrogen |
| GFAP | - | Mouse monoclonal | ab7806 | Abcam |
| GAPDH | - | Rabbit monoclonal | 5174S | Cell Signaling |
| β-actin | - | Mouse (Clone:C4) | 0869100 | MP Biomedicals |
| Anti -Rabbit IgG | HRP | Goat / IgG Polyclonal | 31460 | Thermo Fisher Scientific |
| Anti -Mouse IgG | HRP | Goat / IgG | sc-2031 | Santa Cruz Biotechnology |
| GFAP | - | Rabbit Polyclonal | Z0334 | Dako |
| Olig2 | - | Rabbit Polyclonal | ab42453 | Abcam |
| β3 Tubulin | - | Rabbit Polyclonal | ab76287 | Abcam |
| Anti -Mouse IgG | Alexa Fluor 488 | Goat / IgG Polyclonal | A-21121 | Thermo Fisher Scientific |
| Anti-Rabbit IgG | Cy3 | Goat / IgG Polyclonal | AP132C | Merck Millipore |
