# Supplementary material for: Glioblastoma Stem-Like Cells Are More Susceptible Than Differentiated Cells to Natural Killer Cell Lysis Mediated Through Killer Immunoglobulin-Like Receptors–Human Leukocyte Antigen Ligand Mismatch and Activation Receptor–Ligand Interactions
Source: Front Immunol. 2018 Jun 18;9:1345. doi: 10.3389/fimmu.2018.01345 (PMC6015895; doi:10.3389/fimmu.2018.01345)
Supplement: Supplementary file 3 [file Table_2.docx]

**Supplementary Table II. GBM patient’s biopsies information.**

| Patient ID | Cancer type | Gender | Age | Passage number |
| --- | --- | --- | --- | --- |
| P3 | GBM | Male | 64 | 15-30 |
| 2012-018 | GBM Gliosarcoma | Male | 81 | 5-16 |
| BG5 | GBM | Female | 72 | 13-31 |
| BG7 | GBM | Female | 69 | 28-32 |
| GG1 | GBM | Female | 37 | 5-10 |
| GG9 | GBM | Female | 64 | 10-15 |
