# Supplementary material for: Glioblastoma Stem-Like Cells Are More Susceptible Than Differentiated Cells to Natural Killer Cell Lysis Mediated Through Killer Immunoglobulin-Like Receptors–Human Leukocyte Antigen Ligand Mismatch and Activation Receptor–Ligand Interactions
Source: Front Immunol. 2018 Jun 18;9:1345. doi: 10.3389/fimmu.2018.01345 (PMC6015895; doi:10.3389/fimmu.2018.01345)
Supplement: Supplementary file 4 [file Table_3.docx]

**Suppl. Table III. Phenotype of GBM cells (MFI of markers) in NB and DMEM medias.**

|  | NB | | | | DMEM | | | |
| --- | --- | --- | --- | --- | --- | --- | --- | --- |
|  | P3 | 2012-018 | BG7 | BG5 | P3 | 2012-018 | BG7 | BG5 |
| GFAP | 916 | 1642 | 2444 | 5516 | 2869 | 4832 | 33278 | 8684 |
| vimentin | 538 | 309 | 1188 | 1572 | 333 | 850 | 1375 | 481 |
| nestin | 8554 | 2951 | 2276 | 12942 | 1945 | 10521 | 6605 | 23690 |
| CD15 | 891 | 929 | 574 | 451 | 614 | 822 | 301 | 906 |
| CD133 | 1080 | 897 | 926 | 929 | 1148 | 1917 | 1521 | 2416 |
| A2B5 | 163 | 446 | 206 | 264 | 687 | 1829 | 313 | 719 |
| CD31 | 598 | 471 | 374 | 338 | 279 | 371 | 888 | 868 |
| ULBP-1 | 853 | 835 | 281 | 385 | 694 | 2065 | 969 | 6548 |
| ULBP-2/5/6 | 3237 | 6155 | 272 | 673 | 2102 | 2924 | 1112 | 6892 |
| ULBP-3 | 3059 | 1781 | 390 | 554 | 825 | 2421 | 1013 | 6274 |
| MICA | 2491 | 3702 | 122* | 1153 | 1532 | 3854 | 1329 | 2485 |
| MICB | 774 | 971 | 996 | 902 | 1429 | 1911 | 1307 | 2596 |
| B7-H6 | 378 | 853 | 211 | 330 | 4896 | 4637 | 1465 | 1171 |
| ICAM-1 | 194 | 389 | 100 | 220 | 2326 | 3373 | 622 | 605 |
| CD112 | 1161 | 1337 | 480 | 716 | 6611 | 5626 | 1930 | 2569 |
| HLA-G | 4552 | 11813 | 127 | 702 | 1909 | 2090 | 256 | 1144 |
| HLA-E | 3476 | 2537 | 122 | 1020 | 1698 | 2527 | 617 | 1083 |
| HLA-A, B, C | 1265 | 1806 | 2646 | 4091 | 15457 | 19724 | 5017 | 7269 |
| HLA-DR, DP, DQ | 125 | 3496 | 109 | 380 | 469 | 1007 | 482 | 4576 |
| HLA-A3 | - | 5348 | 185 | 676 | 981 | 5967 | 1196 | 4076 |
| HLA-Bw4 | 1327 | 10694 | - | - | 6227 | 14347 | - | - |

*Different antibody (MFI not comparable)
